# The Molecular Mechanism of Aerobic Exercise Improving Vascular Remodeling in Hypertension

**DOI:** 10.3389/fphys.2022.792292

**Published:** 2022-02-28

**Authors:** Yinping Song, Hao Jia, Yijie Hua, Chen Wu, Sujuan Li, Kunzhe Li, Zhicheng Liang, Youhua Wang

**Affiliations:** ^1^Institute of Sports and Exercise Biology, School of Physical Education, Shaanxi Normal University, Xi’an, China; ^2^School of Health and Sports, Xi’an Fanyi University, Xi’an, China

**Keywords:** hypertension, aerobic exercise, vascular remodeling, vascular smooth muscle cells, endothelial cells

## Abstract

The treatment and prevention of hypertension has been a worldwide medical challenge. The key pathological hallmark of hypertension is altered arterial vascular structure and function, i.e., increased peripheral vascular resistance due to vascular remodeling. The aim of this review is to elucidate the molecular mechanisms of vascular remodeling in hypertension and the protective mechanisms of aerobic exercise against vascular remodeling during the pathological process of hypertension. The main focus is on the mechanisms of oxidative stress and inflammation in the pathological condition of hypertension and vascular phenotypic transformation induced by the trilaminar structure of vascular endothelial cells, smooth muscle cells and extracellular matrix, and the peripheral adipose layer of the vasculature. To further explore the possible mechanisms by which aerobic exercise ameliorates vascular remodeling in the pathological process of hypertension through anti-proliferative, anti-inflammatory, antioxidant and thus inhibiting vascular phenotypic transformation. It provides a new perspective to reveal the intervention targets of vascular remodeling for the prevention and treatment of hypertension and its complications.

## Introduction

**FIGURE 1:**
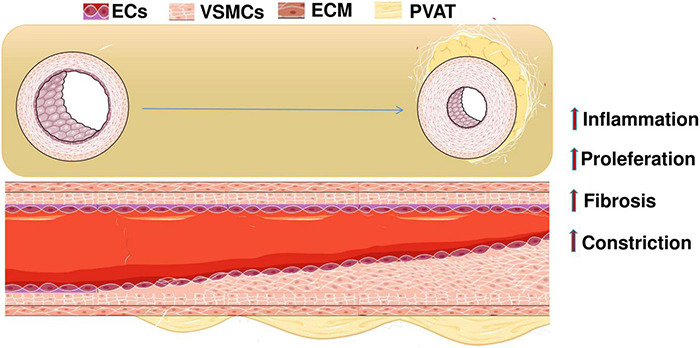
When hypertension occurs, the continuous increase of blood pressure leads to chronic poor vascular adaptation and dysfunction. The specific manifestations are changes in the structure and function of vascular endothelial cells, smooth muscle cells, extracellular matrix, and perivascular adipose tissue. ECs, endothelial cells; VSMCs, vascular smooth muscle cells; ECM, extracellular matrix; PVAT, perivascular adipose tissue.

According to the World Health Organization (WHO), cardiovascular diseases (CVDs) are the number one cause of death worldwide. The number of deaths due to CVDs is expected to rise to approximately 23.2 million in 2030, with cardiovascular deaths accounting for 31% of all global deaths. Hypertension increases patient’s risk of cardiovascular, brain, kidney, and other diseases. WHO recommends 25% relative reduction in prevalence of hypertension in public health targets by 2020 to reduce global disease burden (Diem et al., 2016). Hypertension endangers the health of the vascular system, as evidenced by vascular pathological remodeling. A characteristic pathological alteration of hypertension is augmented vasoconstrictor and attenuated vasodilator responses to various physiological stimuli, resulting in elevated vascular tone in arteries and arterioles that are exposed to persistent high blood pressure. Initially, the vascular remodeling caused by increased blood pressure allows the vasculature to adapt to short-term hemodynamic changes. However, sustained increases in blood pressure leads to chronic vascular maladaptation and dysfunction. This is manifested by structural and functional changes in the vascular endothelium, smooth muscle cells (VSMCs), extracellular matrix (ECM), and perivascular adipose tissue (PVAT) (Figure 1; Ghaffari et al., 2015; Wang and Khalil, 2018).

**FIGURE 2:**
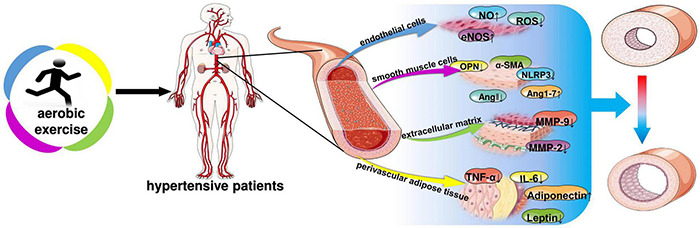
Aerobic exercise improves molecular changes in vascular remodeling of hypertension. NO, nitric oxide; ROS, reactive oxygen species; eNOS, endothelial nitric oxide synthase; OPN, osteopontin; α-SMA, α-smooth muscle actin; NLRP3, NOD-like receptor thermal protein domain associated protein 3; ANG, angiotensin; MMP-9, matrix metallopeptidase 9; MMP-2, matrix metallopeptidase 2; IL-6, interleukin-6; TNF-α, tumor necrosis factor-α; Adiponectin; Leptin.

Hypertension damages blood vessels, which in turn leads to pathological changes in blood vessels—vascular remodeling. In 1994, Gibbons and Dzau introduced the concept of vascular remodeling, which is characterized by vascular dysfunction, vessel wall thickening, and increased wall-to-lumen ratio (Gibbons and Dzau, 1994). Angiotensin II (Ang II), endothelin (ET), nitric oxide (NO), local growth factors (fibroblast growth factor, platelet-derived growth factor, and transforming growth factor beta), and metalloproteinases have been shown to be closely involved in the regulation of hypertension (Brown et al., 2018). Excessive activation of the renin-angiotensin system (RAS) causes diseases such as hypertension. AngII and aldosterone levels lead to vascular fibrosis, inflammation and proliferation. The interaction of oxidative stress and inflammation also leads to vascular remodeling (Schiffrin and Touyz, 2004). United States and European hypertension guidelines encourage regular aerobic exercise in hypertensive patients because of its effectiveness in improving hypertension (Mancia et al., 2007). Aerobic exercise significantly reduces systolic 24-h blood pressure, systolic systemic vascular resistance, and small artery elasticity index (Pagonas et al., 2017). This review summarizes the molecular mechanisms of changes in vascular endothelial cells, smooth muscle cells, extracellular matrix, and vascular peripheral fat during pathological alterations. And further explored the molecular mechanism of aerobic exercise to improve vascular remodeling for the prevention and treatment of hypertension, providing a theoretical basis for the prevention and treatment of hypertension (Figure 2).

## Endothelial Cells and the Areobic Exercise on Vascular Remodeling

Endothelial injury is a critical early step in the development and progression of hypertension. Endothelial damage/repair imbalance causes endothelial dysfunction which in turn induces hypertension. In addition, endothelial cells (ECs) signaling disorders lead to endothelial dysfunction, which is characterized by arterial vascular remodeling (Konukoglu and Uzun, 2017).

### Endothelial Dysfunction

Endothelial cells are seen as the first line of defense between risk factors and vascular disease. Endothelial cells are thought to play an important role in the regulation of local vascular tone. In 1980, Furchgott and Zawadzki (1980) discovered endothelium-derived relaxing factor (EDRF). EDRF is chemically identified as endogenous nitric oxide (NO) (Ignarro et al., 1987). Since then, endothelial dysfunction has become synonymous with reduced NO bioactivity. Furthermore, hemodynamics is ubiquitous and essential physiological stimulus for vascular cells and is thought to exert an important influence on the pathological course of hypertension by regulating endothelial cell function. Shear stress plays a role in the control of endothelial cell proliferation and apoptosis; for example, stable flow reduces EC proliferation, whereas disturbed flow increases EC turnover and stimulates apoptosis (Davies et al., 1986; Akimoto et al., 2000). An increase in shear stress usually causes vasodilation, mostly mediated by an increase in endothelial nitric oxide synthase (eNOS) activity and NO production (Rubanyi et al., 1986; Redmond et al., 1998). Indeed, shear stress is thought to be the primary physiological stimulus for this potent vasodilator molecule. Other endothelium-derived vasoactive substances altered by shear stress include PGI2 (Redmond et al., 1998; Hendrickson et al., 1999) and endothelin-1 (ET-1) (Kuchan and Frangos, 1993; Malek et al., 1993).

Hypertension is associated with endothelial dysfunction (Konukoglu and Uzun, 2017). The main factors of endothelial dysfunction are reduced bioavailability of NO, increased sensitivity of ECs to vasoconstrictors, increased production of vasoconstrictor substances and elevated shear stress (Zhou et al., 2014; Cyr et al., 2020). Bone marrow secretes and releases endothelial progenitor cells (EPCs), which migrate to the peripheral circulation and differentiate into mature vascular endothelial cells (VECs) to maintain vascular integrity. EPC levels are a risk factor for cardiovascular disease and are associated with endothelial endothelium-dependent vasodilation (Vasa et al., 2001). VECs secretes active substances such as NO and ET to maintain vascular homeostasis. In moderate and severe hypertension, VECs damage and imbalance of reactive substances result in decreased NO secretion, increased ET vasoconstrictor, decreased diastolic system function, and vasoconstriction (Iwakiri and Groszmann, 2007).

The nitric oxide synthase (NOS) enzyme catalyzes the eventual production of NO from L-arginine. Mammals have three NOS isoforms: neuronal (nNOS), endothelial (eNOS), and inducible (iNOS). Infection and chronic inflammation induce increased NO production by iNOS. Under hypertensive pathology, increased NO concentration generates reactive nitrogen oxides (RNOS) with oxygen radicals, which indirectly cause apoptosis and tissue damage. In contrast, eNOS, a calcium-dependent protein, has a diastolic effect. Shear stress, acetylcholine, bradykinin, and histamine stimulate eNOS activity and NO production through calcium-dependent and non-dependent way (Zhao et al., 2015). In addition, NO channels are present in the myoendothelial junction (MEJ), a cellular extension that promotes crosstalk connections between endothelial cells and vascular smooth muscle in small arteries and arterioles. eNOS expression in the MEJ limits long-distance diffusion of NO and reduces the scavenging of NO by reactive oxygen species (ROS) (Shu et al., 2019). In addition to targeting eNOS to the MEJ, hemoglobin-α (Hb-α) is enriched in the MEJ by unbiased proteomic screening. Functionally, Hb-α acts as a “NO uptake pool” by buffering NO diffusion from endothelium to smooth muscle cells through the formation of a dioxygenation reaction between nitrate and methemoglobin-α, which further regulates NOS-mediated signaling to control vascular remodeling (Straub et al., 2012). Disruption of eNOS and Hb-α binding with Hb-α mimetic peptide enhances NO signaling and lowers blood pressure *in vivo*. Thereby identifying new targets for the treatment of hypertensive vascular remodeling (Straub et al., 2014).

### Reactive Oxygen Species

Impaired endothelium-dependent vasodilatory function in hypertension is associated with oxidative stress and ROS together with other pathways reduce NO bioavailability (Virdis et al., 2013).

Reactive oxygen species alter gene expression by regulating the activation of transcription factors, with subsequent effects on downstream target proteins, and also regulate the production and degradation of extracellular matrix, inactivate NO function, and stimulate the expression of multiple kinases and pro-inflammatory genes (Monteiro et al., 2019).

Elevated levels of oxidative stress in hypertensive patients lead to an imbalance in the production/accumulation of ROS (Montezano et al., 2015). Nicotinamide adenine dinucleotide phosphate oxidase (Nox) is a major source of ROS in the vascular wall and has been identified as playing a key role in the pathogenesis of hypertension (Magnani and Mattevi, 2019). NOx induces increased ROS production in response to inflammation. In ECs, superoxide reacts with NO to generate peroxynitrite to inhibit oxidative capacity leading to oxidative stress. This further leads to vascular inflammation, fibrosis and remodeling in hypertension (Lopes et al., 2015). In addition, the mechanical forces on the vessel wall are altered in patients with hypertension. Increased stretch leads to endothelial cell proliferation and the release of Interleukin-6 (IL-6), Interleukin-8 (IL-8), ROS, ET, and other pro-inflammatory mediators also contribute to impaired endothelial cell function in hypertensive vessels (Jufri et al., 2015).

### Aerobic Exercise Improves Vascular Remodeling Through Endothelial Cell Regulation

The effect of aerobic exercise on the maintenance of endothelial barrier function is due to the increased heart rate, blood flow and shear stress associated with aerobic exercise, which in turn releases vascular protective molecules, such as NO (Laughlin et al., 2008). This immediately leads to a downregulation of endothelial angiotensin II type 1 receptor expression, which leads to a decrease in nicotinamide adenine dinucleotide phosphate (NADPH) oxidase activity and superoxide anion production, thereby reducing ROS production and maintaining endothelial NO bioavailability (Ramkhelawon et al., 2009). This ultimately allows vasodilation and slows down the vascular remodeling of the hypertensive pathological process.

Aerobic exercise for 16 weeks reduced blood pressure and promoted eNOS expression in 29-week-old rats. And exercise also reduced protein levels of insulin-like growth factor-1 (IGF-1), PI3K, and phosphorylated protein kinase B (p-Akt) (Jufri et al., 2015). Long-term aerobic exercise promotes eNOS expression and reduces hypertension via IGF-1/PI3K/p-Akt pathway (Zhang et al., 2018).

Melatonin (MT) acts as an antioxidant and anti-hypertensive. By activating melatonin receptor 2 (MT2). It can increase ca^2+^ levels in endothelial cells, which in turn plays a key role in activating eNOS to increase NO production and NO bioavailability. Studies have shown that exercise can increase MT levels (Escames et al., 2012). In addition, skeletal muscle hypertrophy induced by exercise training increases the production of follicle-stimulating hormone 1 (Follistatin1, Fstl1) (Escames et al., 2012), which improves the repair of vascular endothelial cell damage and reduces the expression of inflammatory cytokines (Miyabe et al., 2014). Aerobic exercise also induces an increase in eNOS expression and thus improves vascular function by increasing shear force (Suvorava and Cortese-Krott, 2018).

## Aerobic Exercise Improves the Effect of VSMCs on Vascular Remodeling

Vascular remodeling in hypertension is manifested in the midmembrane by a shift from contractile phenotype to synthetic phenotype in VSMCs, which is a hallmark of vascular dysfunction in hypertension (Touyz et al., 2018). Multiple factors such as growth factors, ROS, and mechanical injury have been shown to be involved in VSMCs growth and phenotype conversion (Nishio and Watanabe, 1997; Luo et al., 2012; Hald and Alford, 2014).

### Effects of VSMC-Specific Factors and Signaling Pathway Modulation on Vascular Phenotype Transformation

Vascular endothelium, smooth muscle cells phenotypic transition is regulated by specific factors and signaling pathways such as phosphatidylinositol kinase signaling pathway (PI3K/Akt/eNOS) and mitogen-activated protein kinase cascade reaction (MAPK). VSMC phenotypic features perform functions by virtue of different proteins, such as α-SMA, calreticulin, smooth muscle myosin heavy chain, and SM22α (Zhang et al., 2019). Osteopontin (OPN) and epithelial regulatory proteins are associated with cell growth, synthesis, proliferation, and migration (Seo et al., 2015). Vasoactive stimulation, growth factors and epidermal growth factors are involved in VSMC phenotypic conversion through activation of membrane receptors and intracellular and extracellular signaling pathways (Kennedy et al., 2016). Platelet-derived growth factor-BB (PDGF-BB) binds to PDGF receptors and subsequently activates intracellular signaling cascades such as the protein kinase B (Akt), extracellular signal-regulated kinase (ERK), and p38MAPK pathways (Chen et al., 2015). Akt is a major downstream target of phosphatidylinositol 3-kinase (PI3K). MAPK contains three major members: ERK, p38 MAPK, and c-Jun N-terminal kinase (JNK), of which ERK and p38MAPK are involved in VSMCs phenotype conversion (Ma and Wells, 2014).

### Inflammation Is Involved in VSMCs Phenotype Conversion

Increased concentrations of pro-inflammatory cytokines were observed in smooth muscle cells of hypertensive patients (Chi et al., 2019). Nucleotide-binding oligomerization domain-like receptor protein 3 (NLRP3) inflammatory vesicles activate caspase-1 and thus induce inflammation, thus becoming another new focus for triggering hypertension (Sun et al., 2017).

Nucleotide-binding oligomerization domain-like receptor protein 3forms a complex with atypical squamous cells (ASC) prompting the conversion of procaspase-1 to active caspase-1. Activated caspase-1 prompts the conversion of pro interleukin-1beta (IL-1β) to mature IL-1β ultimately inducing inflammation. Elevated levels of the pro-inflammatory cytokine IL-1β in the vasculature under hypertensive pathology suggest that inflammation is highly associated with hypertensive vascular remodeling (Slaats et al., 2016). Multiple signaling and metabolic dysregulation cause NLRP3 inflammasome activation, such as ca^2+^, ROS, NO, Ang II, and endoplasmic reticulum stress and mitochondrial dysfunction (He et al., 2016). NLRP3 inflammasome activation leads to nuclear factor-kappaB (NF-κB) signaling activation involved in the development and progression of hypertension. NLRP3 gene deletion attenuates Ang II-induced inflammation, VSMC phenotypic transformation and proliferation, and Ang II-induced hypertension and vascular remodeling (Ren et al., 2017).

### Renin-Angiotensin System-Induced Vascular Remodeling in Hypertension

The renin-angiotensin system (RAS) regulates vascular tone and plays a key role in vascular remodeling (Schiffrin, 2012). The RAS consists of series of enzymatic reactions culminating in the generation of AngII in plasma as well as in cardiovascular system. The Ang II/AT1 signaling has been shown to be aberrantly activated in vascular hypertrophy and remodeling by promoting VSMC growth, transdifferentiation and proliferation, eliciting a variety of biological actions of the RAS in the vascular homeostasis (Thomas et al., 2005; Mehta and Griendling, 2007; Zhong et al., 2010; Jin et al., 2012). As a specific Ang II-degredating enzyme, ACE2 suppresses VSMC proliferation and vascular hypertrophy. Loss of ACE2 led to vascular proliferation and elevated migration of SMC while ACE2 overexpression inhibited vascular proliferation and hypertrophy by preventing aortic wall thickening (Strawn et al., 1999; Landon and Inagami, 2005; Ferreira et al., 2009; Zhang et al., 2009; Zhong et al., 2011; Jin et al., 2012; Patel et al., 2012). Excessive activation of RAS under hypertensive pathology causes upregulation of the classical pathway action of the Ang-converting enzyme ACE/Ang II/Ang type I receptor (AT1R) and impairs the protective effect of the ACE2/Ang 1–7/Mas receptor (MasR) pathway.

Patients with hypertension present with locally or systemically elevated Ang II levels, i.e., excessive activation of the classical pathway. Renin released from the kidney converts angiotensinogen (AGT) produced by the liver to Ang I, which is converted to Ang II by the action of Ang converting enzyme (ACE) (Li et al., 2017). Other enzymes may also be involved in Ang II production, such as histones, chymotrypsin, etc. (Passos-Silva et al., 2013). ACE also inactivates bradykinin, which has a vasodilatory effect. The physiological effects of Ang II are mediated by the G protein-coupled receptor family, whose types are type 1 (AT1R) and type 2 (AT2R) (Zhang et al., 2017). Activation of the ACE/Ang II/AT1R pathway stimulates vasoconstriction, sympathetic activation and ROS production, and triggers harmful effects such as endothelial dysfunction, inducing vascular inflammation, thrombosis, proliferation, and fibrosis (Kawai et al., 2017). In contrast, AT2R exerts histoprotective effects, including vasodilatation, anti-inflammatory, and anti-proliferative (Santos et al., 2018).

ACE2 hydrolyzes AngI to produce Ang1-9, which is cleaved by ACE to produce Ang 1–7 (Santos et al., 2018). Ang 1–7 mainly acts through ACE2. Ang 1–7 binds to the specific receptor MasR, a G protein-coupled receptor that triggers anti-inflammatory, anti-fibrotic and anti-proliferative and produces protective effects (Rodrigues Prestes et al., 2017).

### ROS Participates in the Phenotypic Transition of Hypertensive VSMCs

Disruption of ROS signaling leads to the development of several diseases, such as hypertension. In hypertension, Ang II, NE, and ET-1 activate receptors located on the cell membrane, namely AT1, α-AR, and ET receptors. These receptors are coupled to G proteins and activate NADPH oxidase. Activated NADPH oxidases produce ROS, which in turn activate cellular phosphorylation pathways: MAPK, PI3K/Akt. Activated phosphorylation pathways activate transcription factors, such as activator protein-1 (AP-1), p53, NF-κB, and nuclear E2-related factor 2 (Nrf2), which promote post-entry gene transcription into the nucleus of the cell. These target genes encode proteins that subsequently mediate changes in cellular phenotypes, such as hypertrophy, inflammation, necrosis, and apoptosis (Das et al., 2018).

Although cells of different systems perform different functions, redox signaling is very similar. NADPH oxidase is a major source of ROS in endothelial cells, vascular smooth muscle cells, cardiomyocytes, renal cells, and cardiovascular neurons (Nowak et al., 2018). Ang II is an important activator of NADPH oxidase and a stimulator of ROS (Kang et al., 2019). ROS are produced through mechanical stress stimulation of vascular smooth muscle cells, and ROS act through MAPK production to cause cell proliferation, hypertrophy and apoptosis (Gusan and Anand-Srivastava, 2013).

### Aerobic Exercise Improves Smooth Muscle Vascular Remodeling

The powerful stimuli generated by aerobic exercise are associated with vascular remodeling (Green, 2009; Green et al., 2017). Small arteries are the main resistance vessels that regulate flow to different tissues of the body and control blood pressure. Phenotypic conversion of VSMC in these vessels plays an important role in structural remodeling and can lead to various cardiovascular diseases, including hypertension (Owens et al., 2004).

Exercise induced the VSMCs of SHR to maintain a more contractile phenotype, with differentiation protein α-SM-actin and OPN, which is involved during VSMC migration and proliferation and as dedifferentiation marker being inhibited (Chaulet et al., 2001; Speer et al., 2002; Ye et al., 2009; Jiang et al., 2014).

After 8 weeks of aerobic exercise, the phenotype of spontaneously hypertensive rats was reversed, showing an increase in contractile protein expression and a decrease in synthetic protein expression. 12-week aerobic exercise increased the expression of eNOS protein in 3-month-old hypertensive rats, and decreased the expression of ERK and p38, thereby improving VSMC function. Aerobic exercise has a beneficial effect on vascular phenotyping by regulating the balance of Akt and MAPK signal pathways in VSMC. Aerobic exercise enhances the effect of PI3K/Akt/eNOS signaling pathway in normal rats, and maintains a good contractile phenotype of normal rat VSMC (Zhang et al., 2019). Aerobic exercise moves the role of RAS to the protective pathway in several disease models such as hypertension (ACE2/Ang 1–7/MasR) (Frantz et al., 2017). Eight weeks of aerobic exercise inhibits the activity of NF-κB p65, reduces the increase of norepinephrine, epinephrine and the expression of IL-1β and TNF-α in plasma (Qi et al., 2019).

Therefore, aerobic exercise is an effective intervention for hypertensive vascular remodeling. Aerobic exercise is involved in improving the vascular remodeling caused by vascular media injury in many aspects, such as reducing inflammation and activating the protective pathway of RAS from the specific signaling pathway.

## Hypertensive Extravascular Membrane and the Ameliorative Effect of Aerobic Exercise

Adventitial fibroblast (AF) is the main cellular component of the adventitia of blood vessels. Under the pathology of hypertension, the ability of proliferation and migration is enhanced, and a variety of cytokines are secreted, which participates in inflammation and vascular remodeling (Qi et al., 2019). When adventitia fibroblasts are pathologically damaged, ECM is secreted to participate in vascular remodeling. Excessive accumulation of collagen will increase the stiffness of blood vessels and accelerate the development of hypertension. In addition, ECM induces cell signals to regulate cell adhesion, proliferation, migration, and differentiation, and participates in the remodeling of hypertensive blood vessels, among which matrix metalloproteinases (MMPs) are the key factors leading to vascular maladaptation (Castro and Tanus-Santos, 2013; Hua and Nair, 2015). Gelatinase MMP-2 and MMP-9 are vascular disease-related proteins, which are involved in oxidative stress and cause cardiovascular dysfunction, and are involved in vascular remodeling in chronic maladaptive hypertension (Belo et al., 2015; Han et al., 2019).

Biologically active peptides, hemodynamics and reactive oxygen species regulate the expression and activity of MMP-2. Increased MMP-2 can cause poor vascular adaptability due to hypertension (Hardy et al., 2018). MMP-2 stimulates VSMC to interact with the newly formed ECM. ECM triggers intracellular signal transduction through integrin to induce phenotypic transition and continuous migration. VSMC changes from a contractile phenotype to a synthetic phenotype, leading to vascular remodeling under the pathology of hypertension. The tissue matrix metalloproteinase inhibitor TIMP is a secreted protein that can inhibit the activity of MMPs. AF-derived TIMP1 acts on the smooth muscle cells and inflammatory cells in the vascular part through paracrine, inhibiting the enzymatic activity of MMP-9, leading to increased synthesis and secretion of collagen in blood vessels. The expression of Ang II increases during hypertension. Ang II induces the expression and secretion of type I collagen in cultured adventitia fibroblasts (Somanna et al., 2016; Fu et al., 2018). Ang II regulates the expression of MMP-2 and TIMP1 in adventitia fibroblasts, and the changes in the expression of MMP-2 and TIMP1 are involved in the secretion of collagen by adventitia fibroblasts to participate in the process of vascular remodeling.

### ROS Is Involved in the Regulation of Matrix Metalloproteinases

Researches have shown that ROS can regulate the activity of MMPs. Pro-MMP-2 and pro-MMP-9 secreted by VSMC are activated by ROS (Prado et al., 2018). The expression of MMPs genes is also regulated by ROS. When VSMCs are mechanically stretched, NAD(P)H oxidase-derived ROS increases the expression of MMP-2 mRNA (Yue et al., 2018). The strategy of adjusting the bioavailability of ROS can reverse vascular remodeling, effectively prevent vascular damage and reduce hypertension and its related end-organ damage (Prado et al., 2018).

### Ameliorative Effect of Aerobic Exercise

Twelve weeks of exercise training increased collagen deposition in hypertensive rats, and reduced the size of pores in the intima, which explained the beneficial effects of exercise on vascular remodeling and vasodilation, especially the pressure exerted by elastin protein at low positions. The latest research on the aorta of hypertensive rats also shows that exercise training can normalize changes in the deposition of elastic components (Moraes-Teixeira Jde et al., 2010). The imbalance between synthesis and degradation of ECM protein can affect vascular remodeling. Sports training affects the expression of MMP to varying degrees. Under pathological conditions, ROS production will increase ECM proteins, such as collagen and fibronectin (Lee and Griendling, 2008). In addition, the reduction of oxidative stress in hypertension is related to the normalization of vascular remodeling and collagen deposition observed in arteries (Zhang et al., 2016).

## Perivascular Adipose Tissue and the Ameliorative Effect of Aerobic Exercise

### PVAT Adipose Tissue Is Involved in Vascular Remodeling of Hypertension

Perivascular adipose tissue secretes a large number of metabolically vasoactive adipokines (e.g., lipocalin, leptin, resistin, endolipin, etc.) that exert endocrine and paracrine effects (Saxton et al., 2019). Vascular injury, infection leads to abnormal PVAT and inflammatory cell infiltration and imbalance in the release of harmful and beneficial adipokines. This is usually manifested by increased levels of leptin and decreased levels of adiponectin (Zhang et al., 2016). This in turn accelerates inflammation, oxidative stress causing endothelial dysfunction and VSMC proliferation.

The adipokines produced by PVAT are more likely to cause inflammation, proliferation, and then cause vascular remodeling (Schlich et al., 2013; Nosalski and Guzik, 2017). PVAT dysfunction activates the NLRP3/IL-1 signaling pathway after early vascular injury, leading to increased proliferation and differentiation of AF, thereby aggravating vascular adventitia remodeling. PVAT causes endothelial dysfunction by increasing the oxidative stress derived from NADPH oxidase and increasing the production of pro-inflammatory adipokines (such as leptin) (Gil-Ortega et al., 2014). The increase of tumor necrosis factor-α (TNF-α) gene expression in PVAT under hypertension is related to the increase of ET-1 and endothelin receptors. Increased TNF-α gene expression is related to NOS uncoupling and reduced NO release (Virdis et al., 2015). Under the pathology of hypertension, PVAT secretes a large amount of adipokines to accelerate inflammation and oxidative stress, aggravate vascular endothelial dysfunction and VSMC proliferation to accelerate vascular remodeling. Adipose tissue contains AGT and ACE, and the gene expression of AT1 receptor in PVAT is higher (Mikolajczyk et al., 2019). Systemic infusion of Ang II can cause local PVAT inflammation and participate in vascular remodeling of hypertension. Adiponectin induces AMP-activated protein kinase (AMPK) phosphorylation, inhibits the migration of mouse outer membrane fibroblasts and inhibits the expression of nitric oxide synthase (Ghantous et al., 2020).

### Aerobic Exercise Regulating Vascular Remodeling by Ameliorating PVAT

Aerobic exercise can significantly reduce the serum leptin level in PVAT in patients with hypertension and improve leptin resistance, and the adiponectin content increases. Aerobic exercise can improve the low-grade inflammation in obese people and reduce the level of plasma inflammatory cytokines (Sousa et al., 2019).

The activation of endothelial cell mechanical sensors during aerobic exercise stimulates the production of eNOS and NO, reduces vascular oxidative stress, increases antioxidant response and improves NO bioavailability (Sponton et al., 2017; Ruegsegger and Booth, 2018). In addition, aerobic exercise changes the metabolic phenotype of adipose tissue and inhibits the expression of inflammatory markers (Boa et al., 2017). Aerobic exercise is beneficial to restore eNOS activation or reduce iNOS protein expression, both of which are related to the normalization of contractile vascular reactivity in obese rats (Araujo et al., 2018).

Exercise training reduces PVAT inflammation (Lee et al., 2016). Aerobic exercise training stimulates angiogenesis in adipose tissue, improves blood flow and reduces hypoxia and macrophage infiltration (You et al., 2013). It can also prevent or weaken the infiltration of immune cells into PVAT, thereby improving blood vessel function (Boa et al., 2017). At the same time, mechanical stimulation of exercise plays a basic role in preventing endothelial dysfunction by reducing ROS and increasing the bioavailability of NO. Exercise training increases the expression of eNOS protein in the aorta and prevents the up-regulation of iNOS in PVAT. Aerobic exercise also increases the expression of Mn-SOD protein in PVAT and reduces tissue ROS production (Huang et al., 2018).

## Conclusion

**FIGURE 3:**
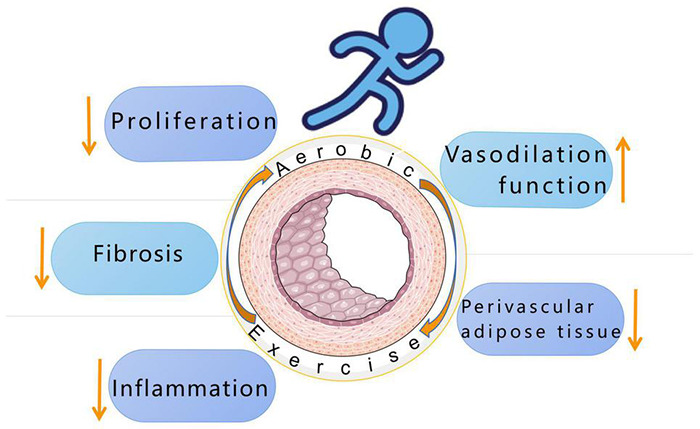
Aerobic exercise improves hypertension by reducing inflammation, reducing fibrosis and proliferation, mediating vasodilation, and reducing perivascular adipose tissue.

To sum up, the pathological changes of the three-layer membrane structure of blood vessels and the increase of perivascular adipose tissue are the factors that lead to the development of hypertensive vascular remodeling. At present, clinically, antihypertensive drugs that may have a beneficial effect on vascular remodeling are being explored, such as neutral lysozyme inhibitors related to angiotensin receptor blockers, aldosterone synthase inhibitors, and renal denervation and baroreceptors Stimulate and other new drugs. In terms of exercise, it has been proven that aerobic exercise can improve vascular remodeling by improving the tunica intima, media, and adventitia thickening and fibrosis under the pathology of hypertension (Figure 3). Based on a large number of previous studies, the future research direction of aerobic exercise and hypertension can be as follows: (1) To further accurately grasp the exercise intensity and exercise time of people of different ages, races and degrees of vascular remodeling. (2) Regular aerobic exercise can reduce ROS in cells and increase the bioavailability of NO, but the mechanism of endothelial function improvement during exercise has not been fully elucidated. Or the protective effect of aerobic exercise in regulating DNA methylation on the cardiovascular system can be used as a further research direction.

## Author Contributions

YS wrote the manuscript. HJ and YH designed the figures along with YS. CW, ZL, SL, and KL reviewed the manuscript writing. YW supervised the manuscript writing and figure making processes. All authors contributed to the article and approved the submitted version.

## Conflict of Interest

The authors declare that the research was conducted in the absence of any commercial or financial relationships that could be construed as a potential conflict of interest.

## Publisher’s Note

All claims expressed in this article are solely those of the authors and do not necessarily represent those of their affiliated organizations, or those of the publisher, the editors and the reviewers. Any product that may be evaluated in this article, or claim that may be made by its manufacturer, is not guaranteed or endorsed by the publisher.
